# Structural Elucidation of a Protective B Cell Epitope on Outer Surface Protein C (OspC) of the Lyme Disease Spirochete, Borreliella burgdorferi

**DOI:** 10.1128/mbio.02981-22

**Published:** 2023-03-28

**Authors:** Michael J. Rudolph, Simon A. Davis, H. M. Emranul Haque, David D. Weis, David J. Vance, Carol Lyn Piazza, Monir Ejemel, Lisa Cavacini, Yang Wang, M. Lamine Mbow, Robert D. Gilmore, Nicholas J. Mantis

**Affiliations:** a New York Structural Biology Center, New York, New York, USA; b Department of Chemistry, University of Kansas, Lawrence, Kansas, USA; c Division of Infectious Diseases, Wadsworth Center, New York State Department of Health, Albany, New York, USA; d MassBiologics of the University of Massachusetts Chan Medical School, Boston, Massachusetts, USA; e Division of Vector Borne Diseases, National Center for Emerging and Zoonotic Infectious Diseases, Centers for Disease Control and Prevention, Fort Collins, Colorado, USA; University of California, Irvine

**Keywords:** *Borreliella burgdorferi*, Lyme disease, X-ray crystallography, monoclonal antibodies, spirochetes, vaccines

## Abstract

Outer surface protein C (OspC) plays a pivotal role in mediating tick-to-host transmission and infectivity of the Lyme disease spirochete, Borreliella burgdorferi. OspC is a helical-rich homodimer that interacts with tick salivary proteins, as well as components of the mammalian immune system. Several decades ago, it was shown that the OspC-specific monoclonal antibody, B5, was able to passively protect mice from experimental tick-transmitted infection by B. burgdorferi strain B31. However, B5’s epitope has never been elucidated, despite widespread interest in OspC as a possible Lyme disease vaccine antigen. Here, we report the crystal structure of B5 antigen-binding fragments (Fabs) in complex with recombinant OspC type A (OspC_A_). Each OspC monomer within the homodimer was bound by a single B5 Fab in a side-on orientation, with contact points along OspC’s α-helix 1 and α-helix 6, as well as interactions with the loop between α-helices 5 and 6. In addition, B5’s complementarity-determining region (CDR) H3 bridged the OspC-OspC′ homodimer interface, revealing the quaternary nature of the protective epitope. To provide insight into the molecular basis of B5 serotype specificity, we solved the crystal structures of recombinant OspC types B and K and compared them to OspC_A_. This study represents the first structure of a protective B cell epitope on OspC and will aid in the rational design of OspC-based vaccines and therapeutics for Lyme disease.

## INTRODUCTION

Lyme disease (LD), or Lyme borreliosis (LB), is the most common tick-borne infection in North America and Europe. Clinical manifestations commonly associated with LD include erythema migrans, neuroborreliosis, carditis, and Lyme arthritis (1, 2). In North America, the primary etiological agent of LD is the spirochete Borreliella burgdorferi, while in Eurasia LD is caused by related genospecies, including B. afzelii and B. garinii (3, 4). In the United States, B. burgdorferi is vectored by blacklegged ticks, Ixodes scapularis and Ixodes pacificus. Naive tick larvae acquire B. burgdorferi during a blood meal on an infected reservoir species, including birds and an array of small mammals. Once within a tick, the spirochetal bacteria remain dormant in the midgut until a second blood meal taken during the nymphal stage, after which B. burgdorferi migrates to the salivary glands, where it is deposited into the skin of an impending host. Perpetuation of B. burgdorferi’s enzootic cycle and persistence within vertebrate reservoirs is contingent on the spirochete’s ability to evade innate and adaptive immune responses through a myriad of mechanisms, including complement resistance, antigenic diversification, antigenic variation, and redundant adhesins and colonization factors (5–8).

With an estimated 476,000 individuals treated for Lyme disease per year in the United States (9), there is an acute need for vaccines that prevent B. burgdorferi transmission and limit human infections (10–12). An LD vaccine known as LYMErix was formerly approved for use in the United States but discontinued in 2002 for a variety of reasons (10, 12, 13). That vaccine, which consisted of recombinant outer surface protein A (OspA), elicited antibodies that blocked transmission of B. burgdorferi by interfering with egress of the spirochete from the tick midgut during the course of a blood meal (14–17). Second-generation OspA-based vaccines are currently in development and clinical trials (18, 19). One shortcoming of these vaccines is that once an infection is established, OspA antibodies are of little consequence, as surface expression of OspA is downregulated by B. burgdorferi within its mammalian host (20, 21).

Outer surface protein C (OspC; BBB19) has long been considered as another candidate LD vaccine antigen. OspC is an ~23-kDa helical-rich lipoprotein expressed by B. burgdorferi in the tick midgut during the course of a blood meal (22–27). OspC facilitates migration of B. burgdorferi from the midgut to the salivary glands and contributes to early stages of mammalian survival (22, 26, 28, 29). In the mouse model, OspC antibodies elicited through active or passive vaccination prevent tick-mediated B. burgdorferi infection (30, 31). Indeed, reminiscent of OspA, recent evidence indicates that OspC antibodies entrap B. burgdorferi within the tick midgut by a mechanism that does not involve direct spirochete killing (32). OspC antibodies also protect mice against B. burgdorferi needle infection and have been reported to promote resolution of arthritis and carditis in a SCID mouse model of Lyme disease (30, 33–37). Indeed, OspC-specific antibodies are associated with serum borreliacidal activity from early LD patients (38, 39). Thus, OspC antibodies afford a double layer of protection by interfering with B. burgdorferi tick-to-host transmission and promoting clearance of spirochetes that gain access to a mammalian host.

A potential drawback of OspC-based vaccines is that OspC is highly polymorphic within and across B. burgdorferi genospecies. At least 25 different *ospC* alleles or OspC types (when referring to the protein) have been identified to date, with extensive diversity occurring even within B. burgdorferi isolates within limited geographical regions (40–45). The degree of variability is such that antibody responses to a given OspC type have limited cross-reactivity with other OspC types (32, 33, 37, 46). Moreover, susceptibility to B. burgdorferi reinfection as well as repeated episodes of LD has been linked to OspC variability and immunodominance (30, 32, 33, 38, 41, 47–49). Thus, a vaccine based on a single OspC type would have limited utility in regions where multiple B. burgdorferi OspC types coexist.

To overcome this challenge, investigative teams have employed multiple sequence alignments, linear B cell epitope analysis, and computational modeling to localize conserved and type-specific residues on OspC associated with protection (41, 42, 50–55). This approach has proven fruitful, as Marconi and colleagues have developed multivalent chimeric vaccines for veterinary use that consist of conserved or minimally variable OspC epitopes from different OspC types (53, 56). In mice, chimeric OspC vaccines elicit broadly reactive antibodies with complement-dependent borreliacidal activity (50, 57, 58). Moreover, a veterinary vaccine consisting of chimeric OspC antigen combined with OspA proved effective at eliciting OspC antibodies and in preventing the onset of LD-like symptoms in dogs following experimental challenge with field-caught B. burgdorferi-infected ticks (56). In a separate line of investigation, Baum and colleagues examined human and mouse immune sera reactivity with a panel of 23 OspC types and delineated residues that determine type-specific cross-reactive antibody binding (41).

In recent years, the emergence of antibody- and structure-based approaches to vaccine design has accelerated the discovery of candidate vaccines for highly antigenically variable pathogens such as influenza virus, HIV-1, SARS-CoV-2, and even malaria (59–61). In effect, antigens are engineered around protective linear or conformational (discontinuous) epitopes defined by high-resolution X-ray and cryo-electron microscopy structures of antigen-antibody complexes. We reasoned that such an approach might be suited to OspC-based vaccine design. Indeed, the structures of three OspC types, A (OspC_A_), I (OspC_I_) and E (OspC_E_), were solved more than 2 decades ago and shown to share a high degree of similarity (Table 1; see Fig. S1 in the supplemental material) (23, 24). OspC consists of four long α-helices (1–3, 6), two shorter α-helices (4 and 5), and two short antiparallel β-strands located between α1 and α2 (23, 24). The biologically functional molecule is a dimer (with monomers referred to here as OspC-OspC′) that assumes a knob-shaped structure anchored via an N-terminal lipidated moiety in the spirochete’s outer membrane (23–25). While the structures of several OspA-antibody complexes have been solved and proved useful in next-generation OspA-based vaccine design (62–64), no OspC-antibody complexes have been reported.

**TABLE 1:** B. burgdorferi OspC crystal structures and PDB codes

Type	Strain	PDB ID	Reference
A	B31	1GGQ	24
B	ZS7	7UJ2	This study
E	N40	1G5Z	85
I	HB19	1F1M	24
K	297	7UJ6	This study

10.1128/mbio.02981-22.1 FIG S1 Structures of OspC types A, I and E. Ribbon diagrams of (A) OspC type A [PDB ID 1GGQ] colored green and cyan; (B) OspC type I [PDB ID 1F1M] colored yellow and magenta; (C) OspC type E [PDB ID 1G5Z] colored light blue and dark green. All alpha-helices (1-6) and beta-strands 1 and 2 are labeled highlighting structural similarity. Download FIG S1, PDF file, 0.3 MB. Copyright © 2023 Rudolph et al. 2023 Rudolph et al. https://creativecommons.org/licenses/by/4.0/ This content is distributed under the terms of the Creative Commons Attribution 4.0 International license.

B5 is an OspC-specific monoclonal IgG2a antibody (MAb) identified in a screen of B cell hybridomas derived from mice infected with B. burgdorferi strain B31 (OspC type A) (65). Passive immunization studies demonstrated that B5 IgG was sufficient to protect mice against experimental tick-mediated B. burgdorferi challenge, possibly by interfering with spirochete egress from the tick midgut (31, 66). Liang and colleagues also demonstrated that passive administration of B5 IgG was sufficient to protect mice against B. burgdorferi challenge by needle injection (67). B5 IgG is used widely as a reagent in the research community and, to our knowledge, remains the only OspC MAb to have been shown to be protective in a tick infection model of the natural route of B. burgdorferi transmission. Thus, we reasoned that deciphering the molecular interactions between B5 IgG and OspC will yield important information about mechanisms involved in blocking B. burgdorferi transmission to and dissemination within mammalian hosts.

## RESULTS

**FIG 1:**
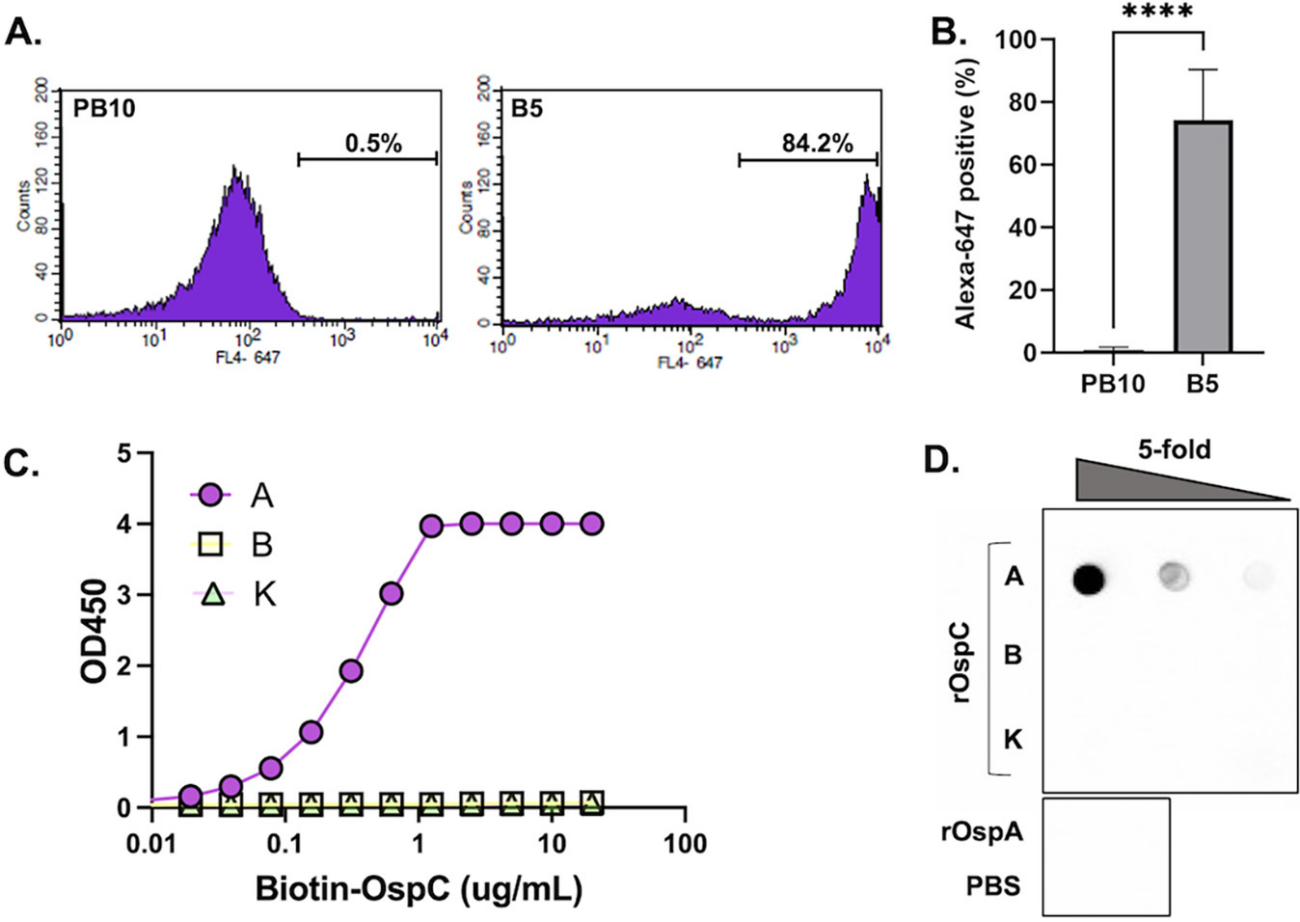
Reactivity of B5 IgG with native and recombinant OspC. (A and B). Mid-log phase B. burgdorferi overexpressing OspC_A_ was incubated with chimeric B5 IgG or an isotype control (PB10) for 1 h at 37°C, washed, and then labeled with Alexa 647-labeled goat anti-human secondary antibody. Cells were analyzed using a BD FACSCalibur flow cytometer. Panel A shows a representative histogram of PB10 (left) and B5 (right) with relative fluorescence plotted on the *x* axis and the number of events plotted on the *y* axis. Panel B shows the total number of Alexa-positive cells following PB10 and B5 treatment from three independent replicates. Asterisks indicate statistical differences (*P* < 0.001) based on Student’s *t* test. (C) Capture of biotin-tagged recombinant OspC types A, B, and K by immobilized B5 IgG, as described in Materials and Methods. The capture ELISA is representative of three independent replicates. (D) Reactivity of B5 IgG with 5-fold serial dilutions of recombinant OspC types A, B, and K spotted onto nitrocellulose membrane. Recombinant OspA (rOspA) and PBS were spotted as negative controls.

B5 IgG was originally isolated from mice that had been experimentally challenged with B. burgdorferi strain B31 (31, 68). Due to a limited supply of hybridoma-derived mouse B5 IgG, we generated a recombinant chimeric derivative of B5 in which the murine V_H_ and V_L_ elements were fused to human IgG_1_ Fc and kappa light chains, respectively, and expressed in Expi293 cells. We confirmed that mouse B5 IgG2a, as well as the chimeric derivative of B5 had reactivity with OspC. Recognition of native OspC on the surface of viable spirochetes was demonstrated by flow cytometry: B. burgdorferi strain B313, which endogenously overexpresses OspC_A_, was incubated with B5 IgG or an isotype control (PB10 IgG) followed by Alexa 647-labeled secondary antibody and then analyzed for mean fluorescence intensity (MFI). B5 IgG labeled 70 to 80% of the live spirochetes with an MFI of >3200, compared to the isotype control, which labeled <1% of cells with an MFI of ~50 (Fig. 1A and B). To assess the breadth of B5 IgG reactivity, enzyme-linked immunosorbent assay (ELISA) plates were coated with recombinant OspC_A_ and OspC type B (OspC_B_) and K (OspC_K_) and then probed with chimeric B5 IgG at a range of concentrations. By ELISA, B5 IgG reacted with OspC_A_ but had no detectable reactivity with either OspC_B_ or OspC_K_ (Fig. 1C). Similar results were observed by dot blot analysis (Fig. 1D). Using biolayer interferometry (BLI), B5 IgG had an apparent dissociation constant (*K*_D_) of ~0.2 nM for recombinant OspC_A_ (Fig. S2). These results confirm that B5 recognizes both native and recombinant OspC_A_ but has no measurable reactivity with OspC_B_ or OspC_K_.

10.1128/mbio.02981-22.2 FIG S2 Representative B5-OspC BLI sensorgram. Biotinylated OspC_A_ (3 μg/mL) in buffer (PBS containing 2% wt/vol BSA) was captured onto streptavidin biosensors and then exposed to 2-fold serial dilutions (100 to 1.56 nM) for 5 min and then 30 min of dissociation, as detailed in Materials and Methods. The results were analyzed using Data Analysis HT version 12.0 software and fit to a 1:2 bivalent analyte model. Download FIG S2, PDF file, 0.03 MB. Copyright © 2023 Rudolph et al. 2023 Rudolph et al. https://creativecommons.org/licenses/by/4.0/ This content is distributed under the terms of the Creative Commons Attribution 4.0 International license.

### B5 IgG epitope localization using hydrogen exchange-mass spectrometry (HX-MS).

**FIG 2:**
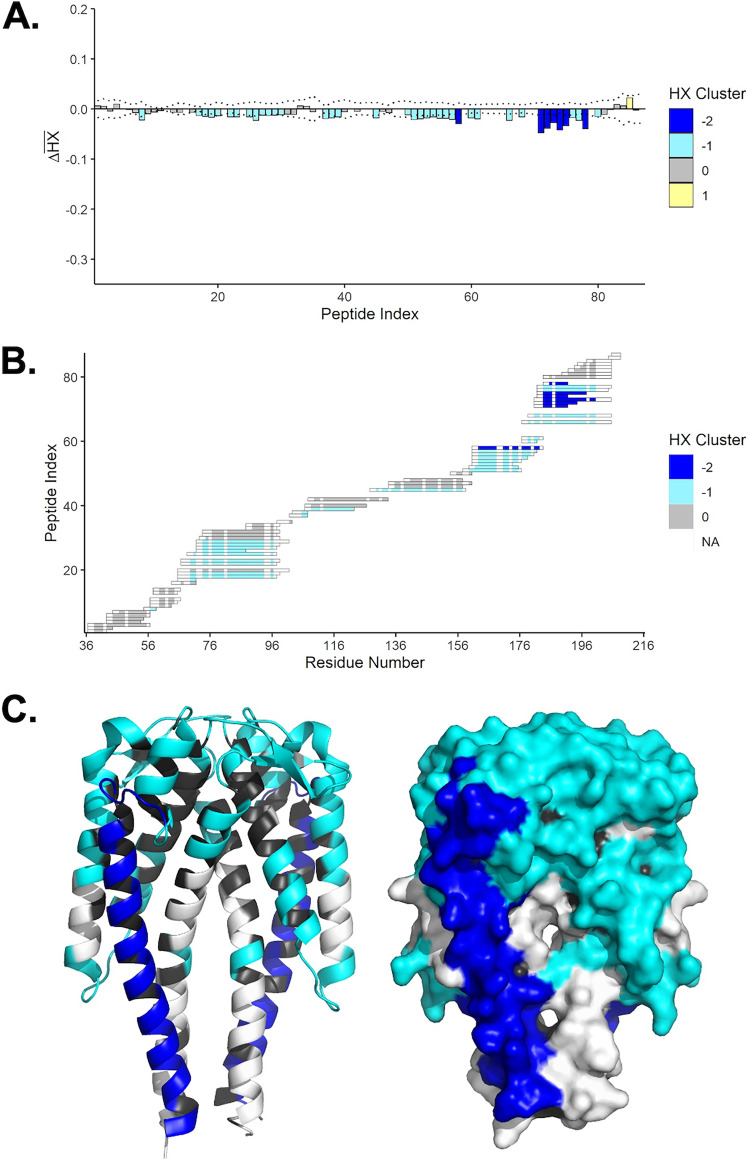
Localization of B5 IgG binding sites on OspC_A_ by HX-MS. (A) Time-averaged, normalized HX differences (Δ*HX*) between OspC_A_ and OspC_A_ bound to B5 IgG. Negative bars denote slower HX in the presence of B5 IgG. The peptide index organizes the peptides from the N to C termini. The values are color-coded based on k-means clustering. Extreme cluster values (e.g., −2) denote stronger effects. (B) Peptide resolution map of the B5 IgG epitope showing clustered HX; (C) Cartoon (left) and surface (right) representations of OspC_A_ [PDB ID 1GGQ] with relative effects of B5 IgG as interpreted from panels A and B. The dark blue shading represents strongly protected regions of OspC_A_, light blue represents weak (but significant) protection, and black denotes lack of coverage.

It was previously reported that B5 IgG recognizes a conformationally sensitive epitope involving the C terminus of α-helix 6 (68). To localize B5 IgG’s epitope in more detail, recombinant homodimeric OspC_A_ was subjected to HX-MS in the absence and presence of B5 IgG. HX-MS provides peptide-level resolution of antibody-antigen interactions in solution, based on differential hydrogen-deuterium exchange rates between unbound and bound targets (69–76). We recently used HX-MS to localize a dozen human B cell epitopes on the B. burgdorferi antigen OspA (77). In the case of OspC, we first generated a peptidic map of OspC_A_, which yielded 87 peptides that covered the entire length of the molecule (Fig. S3). By HX-MS, the N- and C-terminal regions of OspC displayed a high degree of flexibility in the unbound state, as evidenced by a high degree of exchange (data not shown). The addition of B5 IgG resulted in weak, but statistically significant, protection across the majority of the OspC peptides, possibly reflecting a combination of allosteric effects and dynamic interconversion between bound and unbound states of the antigen. The strongest protection, however, encompassed α-helix 6, corresponding to OspC_A_ peptidic residues 163 to 168, 171 to 172, 174 to 175, 177 to 179, 181 to 182, 184 to 186, 188 to 197, and 199 to 200 (Fig. 2). These results demonstrate that binding of B5 IgG influences the flexibility of the entire length of OspC’s terminal α-helix, although HX-MS itself does not afford sufficient resolution to identify the actual antibody-antigen contact points.

10.1128/mbio.02981-22.3 FIG S3 Platformed peptidic map of OspC_A_. OspC_A_ was digested by an in-house-prepared immobilized pepsin column (2.1 by 50 mm). Digested peptides were by trapped and desalted by C_8_ column for 120 s and separated by a C_18_ column. For LC, mobile phase A was 0.1% formic acid in water, and B was 0.1% formic acid in acetonitrile. A total of 25-min LC method, 10 minutes with 15%- to 5% B, was used to separate peptides, as described in Materials and Methods. A total of 87 peptides were identified and mapped for OspC_A_. Download FIG S3, PDF file, 0.08 MB. Copyright © 2023 Rudolph et al. 2023 Rudolph et al. https://creativecommons.org/licenses/by/4.0/ This content is distributed under the terms of the Creative Commons Attribution 4.0 International license.

### X-ray crystal structure of Fab B5-OspC_A_.

**FIG 3:**
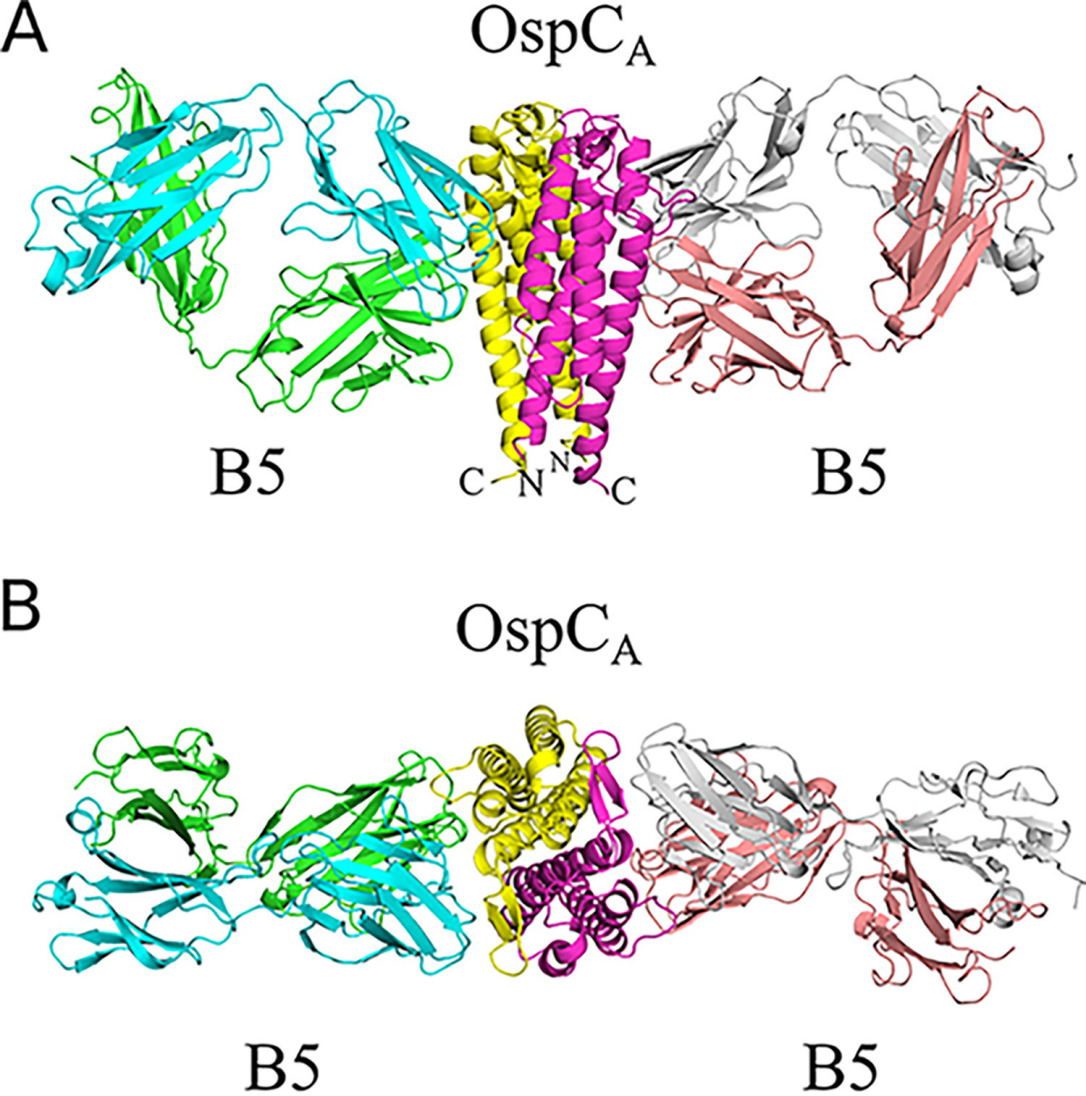
Structure of B5 Fab-OspC_A_. (A and B) Side-on (A) and top-down (B) ribbon diagrams of OspC_A_ homodimer (OspC_A_, OspC_A_′) in complex with B5 Fabs (B5, B5′). The OspC_A_ is colored in yellow and OspC_A_′ is magenta. The B5 Fab V_H_ and C_H_1 elements are colored light green, and the V_L_ and C_L_ are cyan. The B5′ Fab V_H_ and C_H_1 elements are colored in salmon red, and V_L_ and C_L_ are in light gray. The N- and C termini of OspC_A_ and OspC_A_′ are labeled accordingly (N, C).

To define B5’s epitope in greater detail, we solved the X-ray crystal structure of the B5 Fab fragment in complex with OspC_A_ at a 2.7-Å resolution in the P2_1_2_1_2 space group. The crystal structure revealed two B5 Fabs bound to a single OspC_A_ homodimer (1:1 Fab:OspC_A_ stoichiometry) in a side-on fashion (Fig. 3). The B5 Fab fragments (Fab, Fab′) made nearly identical contacts on opposite sides of the OspC_A_ homodimer, as described in detail below. B5 Fabs assumed a canonical structure with two heavy-chain immunoglobulin domains (V_H_, C_H_1) and two light immunoglobulin domains (V_L_, C_L_), each containing seven to ten β-strands arranged in two β-sheets that folded into a two-layer sandwich with all six complementarity-determining regions (CDRs; L1-3, H1-3) on one face of the molecule. The homodimeric structures of OspC_A_ unbound (PDB 1GGQ) and bound to B5 Fabs were similar, as evidenced by a root-mean-square deviation (RMSD) of 1.0 Å for all atoms. Thus, antibody engagement did not induce any major conformational changes in OspC_A_.

**FIG 4:**
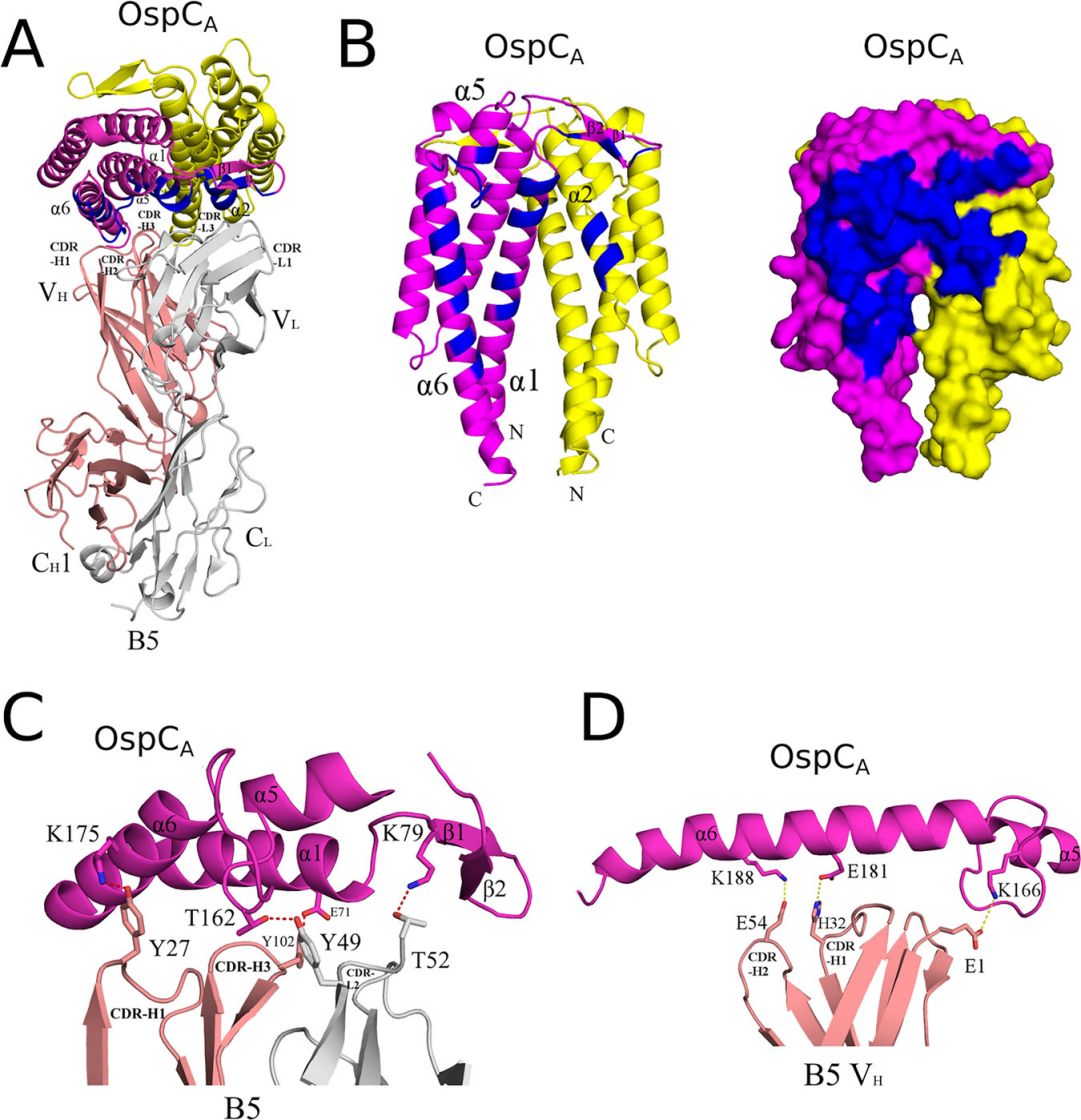
Detailed interactions between B5 and OspC_A_ revealed from the cocrystal structure. (A) Ribbon structure (top-down view) of the OspC_A_ homodimer (OspC_A_, magenta; OspC_A_′, yellow) in complex with a single B5 Fab (V_H_ and C_H_1 elements, salmon red; V_L_ and C_L_, light gray). The OspC_A_ residues that engage with B5 are colored blue. Key secondary structures are labeled (α-helices 1, 2, 5, and 6; β-strands 1 and 2). (B) Ribbon (left) and surface (right) depiction of an OspC_A_ homodimer (OspC_A_, magenta; OspC_A_′, yellow) with B5 interacting residues shaded in dark blue. OspC_A_ N and C termini are labeled N and C, respectively. (C and D) Representations of key (C) H-bonds (red dashes) and (D) salt bridges (yellow dashes) between OspC_A_ (magenta) and Fab B5 (salmon and gray in panel C; gray in panel D). Side chains are drawn as sticks and color coordinated to the main chain color, with nitrogen atoms shaded blue and oxygen atoms shaded red. CDR elements are labeled per convention: CDR-L1, -L2, -L3; CDR-H1, -H2, -H3.

The interaction between B5 Fab and OspC_A_ buried a total surface area of 2,040 Å^2^ (2,002 Å^2^ for the second B5-OspC_A_ interface within the asymmetric unit) establishing 9 hydrogen bonds and 3 salt bridges (12 hydrogen bonds and 3 salt bridges for the second B5-OspC_A_ interface within the asymmetric unit) (Table 2; Fig. 4). The B5 Fab V_H_ domain contributed slightly more to the interaction, burying 1,042 Å^2^ (1,027 Å^2^ for the second B5-OspC_A_ contact within the asymmetric unit) more than the V_L_ domain, which buried 998 Å^2^ (975 Å^2^ for the second B5-OspC_A_ interface in the asymmetric unit). The B5 V_H_ domain formed four hydrogen bonds (three hydrogen bonds for the second B5-OspC_A_ interface in the asymmetric unit), including CDR-H1 residue Tyr-27 with OspC_A_ Lys-175 and CDR-H3 residue Tyr-102 and OspC_A_ Glu-71 (Fig. 4). The B5 V_H_ domain also accounted for two of the three salt bridges observed between B5 and OspC_A._ The two salt bridges formed between B5’s V_H_ domain and OspC_A_ involved H2 Glu-54 with OspC_A_’s Lys-188 and H1 His-32 with Glu-181. The third salt bridge involved V_H_ framework residues Glu-1 and OspC_A_ Lys-166 (Fig. 4). The B5 V_L_ domain formed five hydrogen bonds (eight hydrogen bonds for the second B5-OspC_A_ interface within the asymmetric unit), including CDR L2 Thr-52 with OspC_A_ Lys-79, and CDR L2 Tyr-49 with OspC_A_ Thr-162. There were no salt bridges between the B5 V_L_ domain and OspC_A_.

**TABLE 2:** Summary of OspC_A_-B5 binding data and interface information

Interface	H-bonds^*a*^	Salt bridges	Shape comp.^*b*^	BSA (Å^2^)
1^o^	9	3	0.68	2,040
2^o^^*c*^	12	3	0.61	2,002

a Hydrogen bonds.

b Shape complementarity score.

c Second RTA-V_H_H complex in crystallographic asymmetric unit.

Collectively, the B5 CDR H1, H2, and H3 elements contacted OspC_A_ α-helix 1 and α-helix 6, along with the loop between α-helices 5 and 6 (loop 5-6). The CDR H3 element also buried 30 Å^2^ of α-helix 2′ in the absence of any H-bonds or salt bridges. CDRs L1, L2, and L3 contacted OspC_A_ α-helices 1 and 5, β-strands 1 and 2, and the loop region immediately N-terminal to α-helix 6. CDR L1 and L3 interacted with α-helix 2′, burying 364 Å^2^ and forming a single H-bond between CDR L1 Asn-30 and Gln-110 in OspC_A_. The fact that B5 Fabs straddle the OspC dimer interface not only explains the conformation-dependent nature of B5’s epitope, but more broadly explains the observation that immunizing mice with homodimeric OspC is more effective than monomeric OspC at eliciting protective antibodies (37).

### Structural basis of B5 IgG specificity for OspC_A_.

To elucidate the structural basis for B5 IgG’s specificity for OspC_A_, we solved the crystal structures of recombinant OspC_B_ and OspC_K_ at 1.5-Å and 1.9-Å resolution, respectively, in the P2_1_ space group (Fig. S4). OspC_B_ and OspC_K_ each formed homodimers nearly identical to those of OspC_A_ (unbound or bound to B5). Specifically, OspC_B_ and OspC_K_ monomers each consisted of four long α-helices (1–3, 6), two shorter α-helices (4 and 5), and a two-stranded β-sheet (Fig. S4). The RMSD between the homodimeric OspC_A_ (bound to B5) versus OspC_B_ and OspC_K_ ranged from 0.8 Å to 1.4 Å for all atoms. In each case, the OspC dimer interface is predominantly hydrophobic, with ~80% of the protein atoms in the interface being nonpolar. The monomeric form of OspC_A_ (with or without B5 Fab bound) was structurally more similar to OspC_K_ than OspC_B_, with an RMSD of ~0.7 Å for all atoms. A deletion at residue 74 and an insertion of residue 165 in OspC_B_ relative to OspC_A_ and OspC_K_ accounts for the greater structural deviation of OspC_B_ to OspC_A_ and OspC_K_, as exhibited by an RMSD of 0.9 Å for all atoms when the OspC_B_ monomer was superposed onto OspC_A_ or OspC_K_ monomers. More specifically, atoms in OspC_B_ residues 70 to 75 and 160 to 168 were the most structurally different, with RMSDs of 1.4 Å and 1.9 Å, respectively, compared to the analogous regions in OspC_A_ and OspC_B_. After molecular replacement calculations were performed, the resulting phase information was used to calculate electron density maps utilized to manually insert the correct residues into each model and manually build other regions of each model for the OspC_B_ and OspC_K_ structures. Crystallographic and refinement data for each structure demonstrated a refined molecular model with excellent agreement to the crystallographic data, as well as excellent geometry. B5 V_H_ residues Arg-67 and Ser-77, which have well-defined electron density, are the only two Ramachandran plot outliers (Table S1).

10.1128/mbio.02981-22.4 FIG S4 Structural comparison of OspC_B_ and OspC_K_ with OspC_A_. (A and B) Structures of (A) OspC_B_ (blue and orange), and (B) OspC_K_ (green and slate blue) depicted as ribbon diagrams with the N and C termini labelled. (C) C-α traces of OspC_A_ homodimer from the B5-OspC_A_ complex (magenta and yellow) superpositioned with unbound OspC_A_ (PDB ID: 1GGQ) colored dark gray, OspC_B_ colored medium gray, and OspC_K_ light gray. Download FIG S4, PDF file, 0.3 MB. Copyright © 2023 Rudolph et al. 2023 Rudolph et al. https://creativecommons.org/licenses/by/4.0/ This content is distributed under the terms of the Creative Commons Attribution 4.0 International license.

10.1128/mbio.02981-22.7 TABLE S1 Data associated with crystal structures reported in this study. Download Table S1, PDF file, 0.2 MB. Copyright © 2023 Rudolph et al. 2023 Rudolph et al. https://creativecommons.org/licenses/by/4.0/ This content is distributed under the terms of the Creative Commons Attribution 4.0 International license.

**FIG 5:**
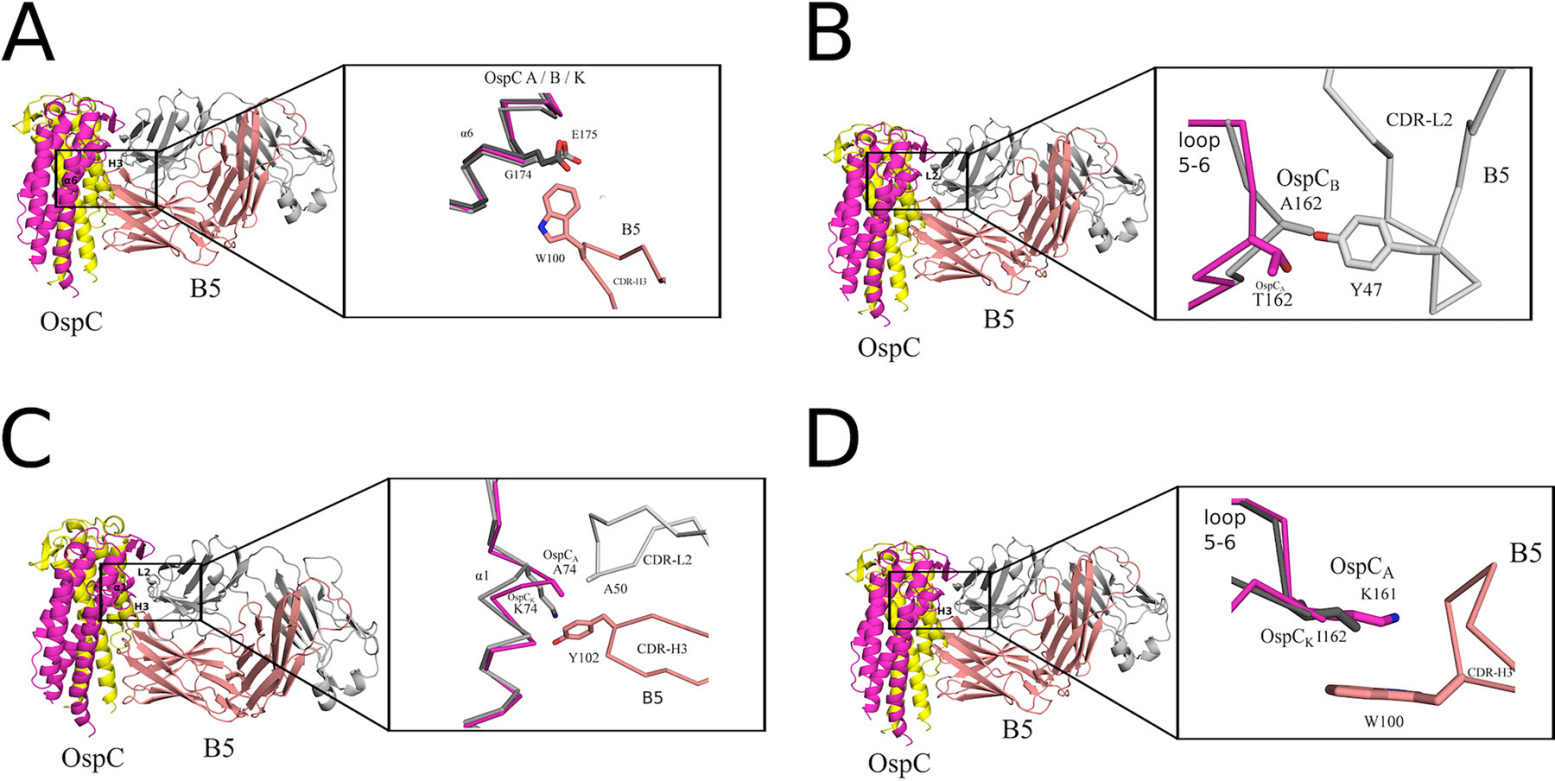
Structural basis of B5 specificity for OspC_A_. Interface between B5 Fab and OspC_A_ superposed with OspC_B_ and OspC_K_ highlighting the key residues in OspC_B_ and Osp_K_ that conceivably disrupt interaction with B5. (A) A zoom-out view of the ribbon diagram of the OspC-B5 complex and a zoom-in view of the C-α traces of OspC_A_ (magenta) bound to B5 (salmon red) with superposed OspC_B_ (light gray) and OspC_K_ (dark gray) highlighting potential electrostatic and steric clash between Glu-175 of OspC_B_ and OspC_K_ with Trp-100 of B5 Fab. (B) The OspC-B5 ribbon diagram with a closeup of the C-α traces of OspC_A_ (magenta) bound to B5 (salmon red) superposed with OspC_B_ (light gray). The image highlights likely repulsion between Ala-162 of OspC_B_ with Tyr-49 of B5 Fab. (C) A zoom-out view of the OspC-B5 complex as a ribbon diagram and a zoom-in perspective of the C-α traces of OspC_A_ (magenta) bound to B5 with CDR-H3 (salmon red) and CDR-L2 (gray) superposed with OspC_k_ (light gray). The image highlights a potential clash between Lys-74 of OspC_K_ and B5’s Tyr-102. (D) The ribbon diagram of the OspC-B5 complex and a closeup of the C-α trace of OspC_A_ (magenta) bound to B5 (salmon red) superposed with OspC_K_ (dark gray). Ile-162 in OspC_k_ precludes a potential π-cation interaction that occurs between OspC_A_ Lys-161 and Trp-100 in B5 Fab. All ribbon diagrams of the OspC-B5 complex are drawn with the OspC dimer colored yellow and magenta. The B5 Fab heavy chain is colored salmon red with the light chain colored light gray. Side chains are drawn as sticks and color coordinated to the main chain color, with nitrogen atoms shaded blue and oxygen atoms shaded red.

Superpositioning the B5-OspC_A_ complex onto OspC_B_ and OspC_K_ revealed additional structural attributes that likely account for B5’s inability to recognize OspC_B_ and OspC_K_. One prominent feature involves the contact between B5 Trp-100 with Gly-174 in OspC_A_. In OspC_B_ and OspC_K_, Glu-175 is superposed with OspC_A_ Gly-174. The bulkier and negatively charged Glu-175 side would be expected to clash (sterically and electrostatically) with Trp-100, thereby impeding B5 interaction (Fig. 5A; Fig. S5). Furthermore, in the case of OspC_B_, an “insertion” of Ala-162 within loop 5-6 relative to OspC_A_ alters the configuration of the loop, resulting in a theoretical clash of B5 with Tyr-47 (Fig. 5B; Fig. S5). OspC_B_ is also unable to H-bond with B5 Tyr-47, due to an Ala rather than a Thr at position 162, as is the case in OspC_A_. The absence of this H-bond donor would be expected to compromise the B5-OspC_B_ interaction. Another structural feature in OspC_B_ disfavoring B5 binding includes deletion of one residue immediately before Lys-74 in OspC_B_. This deletion substantially altered residue positions 73 to 75 within α-helix 1 of OspC_B_ relative to the same residues in OspC_A_ and OspC_K_. As a result, the superposed side chain of Lys-74 in OspC_B_ clashes with B5 Tyr-102. Though several preferred rotamers of OspC_B_ Lys-74 can readily pivot away from B5 Tyr-102, alleviating the close encounter between these two residues, movement of Lys-74 away from Tyr-102 and B5 residue Ala-50 would significantly reduce contact between B5 and OspC_B_ by ~50 Å^2^, likely diminishing B5 binding to OspC_B_ (Fig. 5C; Fig. S5). In the case of OspC_K_, divergent primary amino acid sequences at consequential residues further contributed to a lack of B5 recognition. For example, OspC_A_ Lys161 forms a π-cation interaction with B5 Trp-100, which cannot occur in the case of OspC_K_ due to an Ile residue at this position (Fig. 5D; Fig. S5). To determine the potential cross-reactivity of B5 with the other OspC types, we examined primary sequence conservation across each OspC, focusing on regions in OspC_A_ that support B5 binding while also considering regions in OspC_B_ and OspC_K_ that seemingly antagonize B5 interaction. We predict that two OspC types, C3 and I3, with 76% and 78% overall sequence identities to OspC_A_, respectively, would interact with B5. C3 and I3 possess similar sequences within α-helix 1 and loop 5-6 along with a few other key B5 contact residues found in OspC_A_ (Fig. S6). For example, OspC_I3_ is similar to OspC_A_ in that it has a Gly at position 174, providing ample room to contact B5’s Trp-100. In the case of OspC_C3_, an Asp residue replaces Gly-174; while the Asp side chain is larger than that of Gly, there is still sufficient space to engage Trp-100 in B5. It is interesting that Baum and colleagues demonstrated experimentally that OspC_A_ and OspC_I3_ were the most immunologically cross-reactive pairs in a protein microarray consisting of 23 OspC types (41). Structural insights into the molecular interactions that promote or repel protective antibodies has important implications for rational vaccine design.

10.1128/mbio.02981-22.5 FIG S5 Sequence alignment of OspC A, B, and K. Sequence alignment of OspC types A, B, and K from B. burgdorferi depicting the B5-interacting region of OspC_A_. Magenta asterisks depict B5 V_L_ (L1 to L3) interacting residues, cyan asterisks depict B5 V_H_ (H1 to H3) interacting residues, and blue asterisks denote interaction with B5 V_L_ and V_H_ framework region residues. Secondary structural elements from OspC_A_ bound to B5 are illustrated with α-helices 1 to 6 drawn as black coils and β-strands 1 to 2 drawn as black arrows above the sequence and labelled accordingly. Red background with white letters connotes sequence identity; white background with red letters connotes sequence similarity. The orange box below sequence region 100 to 110 shows B5 contact with the second OspC_A_ monomer. Figure made with ClustalW and ESPript 3.0, as described in Materials and Methods. Download FIG S5, PDF file, 0.3 MB. Copyright © 2023 Rudolph et al. 2023 Rudolph et al. https://creativecommons.org/licenses/by/4.0/ This content is distributed under the terms of the Creative Commons Attribution 4.0 International license.

### Location of functional residues and human linear B cell epitopes on OspC relative to B5’s binding site.

**FIG 6:**
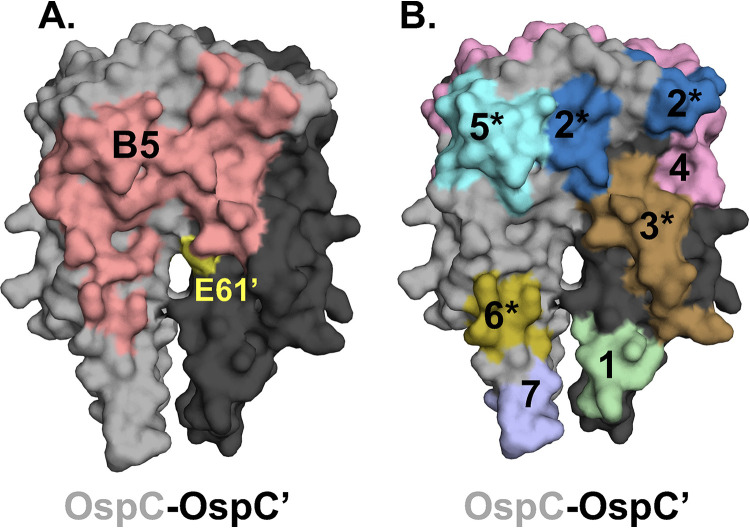
Depiction of functional residues and linear human B cell epitopes and B5 contact points on the OspC homodimer. Surface representation of the OspC_A_ homodimer (PDB 1GGQ) with one monomer colored light gray (OspC) and the other charcoal (OspC′). (A) Depiction of B5’s “footprint” (salmon red) on the OspC dimer. Residue E61 (see the text for details) on OspC′ is colored in yellow. (B) Seven (1 to 7) reported linear B cell epitopes on OspC, as detailed in Table 3. The asterisks (*) indicate linear epitopes that are predicted to fall within B5’s epitope.

OspC is a multifunctional protein that interacts with substrates in both the vector (e.g., Salp15) and mammalian hosts (e.g., plasminogen, C4b) (5). While the exact residues on OspC involved in these interactions have not been elucidated, Eicken and others described a putative ligand binding domain (LBD1) situated at the dimer interface (Fig. 6A) (23). Earnhart and colleagues demonstrated that a point mutation in Glu-61 (E61Q) in proximity to LBD1 of OspC_A_ rendered B. burgdorferi strain B31 noninfectious in a mouse model of needle (subcutaneous) challenge (51). We used PyMol to visualize the location of the OspC_A_ E61 relative to B5’s epitope, with the idea that occlusion of this residue by B5 might account for B5’s functional activity *in vivo* (Fig. 6A). While the interface between B5 Fab and Lys-60, Glu-61, and Glu-63 within LBD1 is minimal (37 Å^2^ in first asymmetric unit; 25 Å^2^ in the second asymmetric unit), B5 V_H_ residues nonetheless engage two residues (Lys-60 and Ala-64) on opposing sides of the LBD1 cavity, as defined by Earnhart and colleagues (51). For that reason, we cannot rule out the possibility that B5 IgG does in fact limit LBD1 ligand accessibility.

To put B5’s epitope in context with other known landmarks on OspC, we extracted the seven human linear B cell epitopes on OspC from the Immune Epitope Database (IEDB.org) (Table 3) and mapped them, using PyMol, onto the crystal structure of dimeric OspC_A_ (Table 3; Fig. 6B). Four of the seven linear epitopes (no. 2, 3, 5, 6) are predicted to fall within B5’s binding site, revealing an overlap between B5’s epitope and known human B cell epitopes on OspC. However, functional activities have not been ascribed to any of these four linear epitopes to date, so we can only speculate as to the impact of antibody occupancy on OspC activity *in vivo*. The two linear B cell epitopes (no. 4, 7) on OspC that have been associated with complement-dependent borreliacidal activity are outside of B5’s footprint (Table 3; Fig. 6B).

**TABLE 3:** Human linear B cell epitopes on OspC and putative overlap with B5 contact points

No.	Residues^*a*^	Sequence	B5^*b*^	IEDB ID^*c*^	Reference
1	38–53	KGPNLVEISKKITDSN	−	559957	96
2	71–86	EIAAKAIGKKIHQNNG	+	12383	97
3	104–118	ISTLIKQKLDGLKNE	+	28749	97
4	130–150	CSETFTNKLKEKHTDLGKEGV	−	6984	98
5	156–171	AKKAILITDAAKDKG	+	181187	99
6	184–190	LKAAKEM	+	560173	96
7	195–210^*b*^	PVVAESPKKP	−	49993	100

a Sequences from OspC type A (strain B31), except 5 from OspC_K_.

b +, Spatial overlap with B5’s epitope; –, No overlap with B5.

c The Immune Epitope Database (IEDB) unique identified number. The underlined numbers in the first column (far left) indicate an epitope associated with borreliacidal activity.

## DISCUSSION

OspC has long been considered a prime LD vaccine antigen due to its vital role in B. burgdorferi pathogenesis, coupled with the fact that it is a target of protective antibodies capable of interfering with both tick-mediated transmission and early stages of mammalian infection (11). Indeed, recombinant (chimeric) antigens with concatenated linear B cell epitopes from different OspC types have proven to be effective veterinary vaccines and may have applications to humans (13, 53). However, linear B cell epitopes constitute only one facet of the antibody response to OspC. There is considerable evidence that conformation-dependent epitopes on OspC are essential in eliciting protection against B. burgdorferi experimental challenge via tick bite and needle injection (37, 68). Despite this fact, a high-resolution map of discontinuous protective and nonprotective B cell epitopes on OspC is lacking.

In this study, we report the first structure of a protective B cell epitope on OspC, as defined by the mouse monoclonal antibody, B5. B5 is the only OspC-specific monoclonal antibody to have been shown to passively protect mice against both needle- and tick-mediated B. burgdorferi infection (31, 78, 79). The B5 Fab-OspC_A_ structure is notable in several respects. First, the structure reveals that B5 attacks the OspC_A_ homodimer at a right angle, favoring an interaction with OspC’s stem (α-helices 1 and 6), rather than the more accessible head (24). While the significance of this side-on orientation is currently unknown, it does result in at least partial occlusion of OspC’s LBD1, which includes residues implicated in B. burgdorferi infectivity of mice (51). Whether occlusion of LBD1 is related to B5 IgG’s ability to limit dissemination of B. burgdorferi in the mouse model remains to be determined.

Second, the B5 Fab-OspC_A_ structure reveals that B5’s epitope is quaternary, as the V_H_ and V_L_ elements span the OspC-OspC′ dimer interface (Fig. 4B). This observation reaffirms the notion that OspC exists as a dimer on the surface of B. burgdorferi, because B5 IgG was isolated from mice that had been infected by tick-bite with viable spirochetes (31, 65). It also explains, at least in part, why monomeric OspC is significantly less effective than dimeric OspC at simulating protective immunity (37). Selection and affinity maturation of B5-like antibodies would only arise in the presence of OspC homodimers in which the OspC-OspC′ interface is preserved. This fact has obvious implications for OspC-based subunit vaccines, as alluded to by others (37). Finally, B5 adds to the growing list of protective monoclonal antibodies that target quaternary epitopes, including on AB toxins (80) and viruses such as Ebola (81) and SARS-CoV-2 (82).

In an effort to define the molecular basis of B5’s specificity for OspC_A_, we also solved the crystal structures of dimeric OspC types B and K (Table 1). The addition of OspC_B_ and OspC_K_ to the list of available structures is significant because B. burgdorferi invasive strains are primarily associated with OspC types A, B, I, and K (42–44). Having all four OspC structures publicly available will enable them to be used for computational-based design of broadly reactive vaccine antigens following a playbook similar that used for influenza virus (83). As predicted, the overall tertiary and quaternary structures of OspC_B_ and OspC_K_ are similar to each other and to the other available OspC structures. Nonetheless, we identified both primary and secondary elements on OspC that likely account for B5 recognition of OspC_A_, but not OspC_B_ or OspC_K_. Unfortunately, the inclusion of computational modeling to assess B5-OspC dynamics and reactivity with other OspC types was beyond the scope of the current study.

As a final note, it is worth pointing out that Eicken and colleagues commented on the similarity of the structure of OspC to that of the variable surface glycoprotein (VSG) from Trypanosoma brucei (84, 85). Twenty years later this parallel can be extended to include comparisons between B5-OspC and VSG-antibody complexes. A recent report describes the structures of three llama-derived single-domain antibodies (nanobodies or VHHs), including one with potent activity against living parasites, including arresting trypanosome motility and promoting membrane blebs (86). Reminiscent of the B5-OspC structure, the authors found that the three nanobodies engage VSG at a right angle near the base of the molecule rather than the head, where they had predicted. Interestingly, the authors argue that engagement with VSG in this orientation has profound effects on trypanosome membrane flexibility, which ultimately impairs the parasite’s motility. A similar mechanism could be at play in the case of B5 and OspC. Occupancy of the lateral face of OspC would be expected to perturb higher-order OspC oligomers or lattices on the bacterial surface, which have been alluded to in the literature (23). Alternatively, B5 might perturb overall membrane fluidity and lipid raft formation critical to B. burgdorferi pathogenesis (87). In theory, any given antibody bound to OspC would be expected to affect OspC fluidity in the spirochete membrane. However, not all OspC monoclonal antibodies are protective (37), so other factors such as epitope specificity may be at play. Sorting out the antibody determinants that influence the outcome between B. burgdorferi and host infectivity ultimately will inform a next-generation LD vaccine.

## MATERIALS AND METHODS

### Mouse and chimeric B5 IgG MAbs.

Lyophilized B5 IgG from the CDC was reconstituted to final concentration of 6 mg/mL. B5 Fab fragments were generated by papain digestion followed by affinity depletion of the Fc fragment by protein A fast protein liquid chromatography (FPLC). The resulting B5 Fab was purified to homogeneity by size exclusion chromatography (SEC) using a Superdex 200 16/60 gel filtration column. The B5 mouse hybridoma was cultured as described previously from frozen aliquot (31). In addition, to ensure sufficient supply of B5 MAb, the mouse B5 V_H_ and V_L_ regions were cloned into human IgG1 Fc and κ light chain expression vectors and used to transfect Expi293 cells following protocols previously described (88). The resulting chimeric B5 IgG1 was purified and used for dot blot and flow cytometry analysis.

### Affinity measurement using biolayer interferometry (BLI).

Biotinylated OspC_A_ (3 μg/mL) in buffer (phosphate-buffered saline [PBS] containing 2% wt/vol bovine serum albumin [BSA]) was captured onto streptavidin biosensors (no. 18-5019, Sartorius, Göttingen, Germany) for 5 min. After 3 min of baseline in buffer, sensors were then exposed to a 2-fold serial dilution of MAb B5, ranging from 100 to 1.56 nM, for 5 min. The sensors were then dipped into wells containing buffer alone for 30 min. An eighth sensor was also loaded with biotinylated-OspC_A_, but not exposed to MAb B5, and was thus used as a background drift control and subtracted from the other sensor data. The raw sensor data were then loaded into the Data Analysis HT 12.0 software, and the data were fit to a 1:2 bivalent analyte model. Data were captured on an Octet RED96e biolayer interferometer (Sartorius) using the Data Acquisition 12.0 software.

### Indirect fluorescent antibody staining and flow cytometry.

B313, a derivative of B. burgdorferi strain B31 that endogenously expresses OspC_A_ as described by Sadziene et al. (89) was kindly provided by Yi-Pin Lin (Wadsworth Center). The strain was cultured in BSK-II medium at 37°C with 5% CO_2_ to mid-log phase. Cells were collected by centrifugation (3,300 × *g*), washed with PBS, resuspended in BSK-II (minus phenol red indicator) at a final concentration of 1 × 10^8^ cells/mL, and incubated at room temperature for 30 min. A total of 5 × 10^6^ cells in 50 μL were incubated with 10 μg/mL of chimeric B5 IgG1 at 37°C for 1 h. Incubation with an isotype control, PB10, was included as a negative control (90). The reaction volume was then increased with the addition of 450 μL of BSK-II (minus phenol red), and goat anti-human IgG [H+L]-Alexa 647 (Invitrogen) was added at a 1:500 dilution and allowed to incubate at 37°C for 30 min. Alexa-647-labeled cells were analyzed on a BD FACSCalibur flow cytometer. Data were obtained and analyzed using BD’s CellQuest Pro software.

### Dot blot analysis.

Recombinant OspC types A, B, and K (Table 1) at 0.5 μg/μL, were 5-fold serially diluted in PBS, and 2 μL of each dilution was spotted on a dry nitrocellulose membrane. The spots were allowed to air dry for 1 h and then were blocked with 5% milk in 1× Tris-buffered saline with Tween 20 (TBS-T) for 18 h and incubated with 0.1 μg/mL chimeric B5 IgG1 in 5% milk in 1× TBS-T at room temperature for 1 h. The membrane was then washed twice with 1× TBS-T, incubated with a 1:10,000 dilution of goat anti-human IgG (H+L)-horseradish peroxidase (HRP; Invitrogen), and washed twice more before detection with enhanced chemiluminescence (ECL; ECL Plus Western blotting substrate; Pierce, Thermo Fisher, Waltham, MA). Images were acquired and analyzed using an iBright 1500 system (Invitrogen).

### OspC ELISA.

B5 IgG was coated onto wells of a 96-well Immulon 4HBX plate (Thermo Fisher, Waltham, MA) at 1 μg/mL in PBS overnight at 4°C. Wells were then blocked for 2 h at room temperature with 2% goat serum in 0.1% Tween 20 in PBS. Biotinylated OspC types A, B, and K were then diluted 2-fold across the plate, starting at 20 μg/mL. Plates were washed, and then captured biotinylated OspC was detected with avidin-HRP (Pierce, Rockford, IL) for 1 h. Plates were washed again, and capture was visualized with SureBlue *N*,*N*,*N′*,*N′*-tetramethyl-1,3-butanediamine (TMB; SeraCare, Milford, MA). The reaction was stopped with 1 M phosphoric acid, and the optical density at 450 nm was read using a SpectraMax iD3 instrument (Molecular Devices, San Jose, CA).

### Liquid chromatography-mass spectrometry (LC-MS) analysis.

For peptic peptide mapping, recombinant OspC_A_ was diluted with quench solution (200 mM glycine, pH 2.5), and 50 pmol of sample was injected in each run. OspC_A_ was digested by an in-house-prepared immobilized pepsin column (2.1 by 50 mm) (91). Digested peptides were trapped and desalted by C_8_ (Zorbax 300SB C_8_, 2.1 by 12.5 mm, 5-μm particles) for 120 s and separated by a C_18_ column (Zorbax 300SB 2.1 by 50 mm, 3.5 μm particle diameter, Agilent, Santa Clara, CA). For LC, mobile phase A was 0.1% formic acid in water, and B was 0.1% formic acid in acetonitrile. A total of a 25-min LC method was used: 10 min with 15% to 35% B was used to separate peptides, and 15 min was used for cleaning purposes. Peptides were detected, and the mass was measured with a quadruple time of flight (Q-TOF) mass spectrometer (Agilent 6530 in electrospray ionization (ESI)-positive ion mode). All the peptic peptides were assigned by tandem mass spectrometry (collision induced dissociation [CID] fragmentation). Agilent MassHunter Qualitative Analysis with BioConfirm (version B.07.00) software was used for the analysis of all the mass spectrometry data. A total of 87 peptides were identified and mapped as shown in Fig. S1. This map shows 100% OspC_A_ sequence coverage with a median length of 17.0 residues and 8.6 average redundancy.

### Hydrogen exchange-mass spectrometry (HX-MS) and data analysis.

OspC_A_ and B5 MAb were buffer exchanged and assayed using our previous protocol (77). Previously flash-frozen OspC_A_ (19 μM) was thawed at room temperature. B5 sample was collated from 4°C and buffer exchanged on the day of experiment. Free the protein state (OspC_A_), sample was prepared by diluting 19 μM OspC_A_ to 9 μM by the addition of 20 mM phosphate and 100 mM NaCl, pH 7.40. The bound state (OspC_A_ +B5) was prepared by three strokes of mixing and adjusted to a final concentration of 9 μM. HX-MS labeling conditions, robot methods, maximally deuterated experiment (OspA paper) protocol, and data analysis were done as recently reported (77).

### Cloning, expression, and purification of OspC.

The PCR amplicon encoding B. burgdorferi OspC_A_ (residues 38 to 201) was subcloned into the pSUMO expression vector that contained an N-terminal deca-histidine and SUMO tag. The PCR amplicons for B. burgdorferi OspC_B_ and OspC_K_ containing residues 38 to 202 were subcloned into the pMCSG7 expression vector that contained an N-terminal deca-histidine tag. Cloning was performed using standard ligase-independent cloning (LIC). All OspC types were expressed in Escherichia coli strain BL21(DE3). The transformed bacteria were grown at 37°C in TB medium and induced at 20°C at an optical density at 600 nm (OD_600_) of 0.6 with 0.1 mM isopropyl-β-d-thiogalactopyranoside (IPTG) for ~16 h. After induction, cells were harvested and resuspended in 20 mM HEPES (pH 7.5) and 150 mM NaCl. The cell suspension was sonicated and centrifuged at 30,000 × *g* for 30 min. After centrifugation, the protein-containing supernatant was purified by nickel-affinity and size exclusion chromatography on an AKTAxpress system (GE Healthcare), which consisted of a 1-mL nickel affinity column followed by a Superdex 200 16/60 gel filtration column. The elution buffer consisted of 0.5 M imidazole in binding buffer, and the gel filtration buffer consisted of 20 mM HEPES (pH 7.5), 150 mM NaCl, and 20 mM imidazole. Fractions containing each OspC type were pooled and subjected to tobacco etch virus (TEV) protease cleavage (1:10 weight ratio) for 3 h at room temperature to remove the respective fusion protein tags. The cleaved proteins were passed over a 1-mL Ni-NTA agarose (Qiagen) gravity column to remove TEV protease, cleaved residues, and uncleaved fusion protein. After purification, Fab B5 was complexed with OspC_A_ in a 1:1 stoichiometry and then concentrated to 10 mg/mL final for all crystallization trials.

### Crystallization and data collection.

All crystals were grown by sitting drop vapor diffusion using a protein to reservoir volume ratio of 1:1 with total drop volumes of 0.2 μL. Crystals of the B5 Fab-OspC_A_ complex were produced at 22°C using a crystallization solution containing 100 mM sodium cacodylate, pH 6.5, 5% polyethylene glycol (PEG) 8K, and 40% Hexylene glycol (MPD). Crystals of the OspC_B_ were produced at 22°C using a crystallization solution containing 100 mM sodium phosphate citrate, pH 4.2; 41.9% PEG 600 crystals of the OspC_K_ were produced at 4°C using a crystallization solution containing 100 mM Tris, pH 8.5, 40% PEG 400, 200 mM LiSO4, and 10 mM 2-aminoethanesulfonic acid. All crystals were flash-frozen in liquid nitrogen after a short soak in the appropriate crystallization buffers supplemented with 25% ethylene glycol. Data were collected at the 24-ID-E beamline at the Advanced Photon Source, Argonne National Labs. All data were indexed, merged, and scaled using HKL2000 (92) and then converted to structure factor amplitudes using CCP4 (93).

### Structure determination and refinement.

The B5 Fab-OspC_A_ complex, OspC_B_, and OspC_K_ structures were solved by molecular replacement using Phaser (92). Molecular replacement calculations were performed using the coordinates of the murine monoclonal Fab 3E6 V_H_ and C_H_1 domains (PDB ID: 4KI5) along with the V_L_ and C_L_ domains of the human germ line antibody hepatitis E virus E2S antibody (PDB ID: 3RKD) as the search model for Fab B5 in the B5-OspC_A_ complex. The OspC coordinates (PDB ID: 1GGQ) were used as the search model for OspC_A_ in the OspC_A_-B5 complex. The same OspC coordinates (PDB ID: 1GGQ) were also used as search models for the OspC_B_ and OspC_K_ structure determinations. The resulting phase information from molecular replacement was used for some manual model building of each structure solved using the graphics program Coot (94) and structural refinement employing the PHENIX package (95). Data collection and refinement statistics are listed in Table S1, as are the Protein Data Bank (https://www.rcsb.org/) codes associated with each of the three structures generated in this study (B5-OspC_A_, PDB ID 7UIJ; OspC_B_, PDB ID 7UJ2; OspC_K_, PDB ID 7UJ6). Molecular graphics were prepared using PyMOL (Schrodinger, DeLano Scientific LLC, Palo Alto, CA). All buried surface area calculations were done with PISA within the CCP4 suite (93).

10.1128/mbio.02981-22.6 FIG S6 Sequence alignment of OspC types that conceivably bind B5. Primary sequence alignment of OspC types C3 and I3 with OspC_A,_ OspC_B_, and OspC_K_ highlighting the key sequence similarities of OspC_C3_ and OspC_I3_ to OspC_A_. Red rectangles encapsulate critical residues in α-helix 1 and loop 5-6 highlighting the insertion at residue 74 and deletion at residue 165 in OspC_B_ that antagonize B5 binding. Red asterisks above the sequence identify the critical OspC_A_ residues 161 and 175, which support interaction with B5. Black asterisks below the sequence denote sequence identity, with two dots and one dot showing relatively reduced sequence similarity. Figure made with Clustal Omega. Download FIG S6, PDF file, 1.5 MB. Copyright © 2023 Rudolph et al. 2023 Rudolph et al. https://creativecommons.org/licenses/by/4.0/ This content is distributed under the terms of the Creative Commons Attribution 4.0 International license.
