# Supplementary figures and images for: Structural Elucidation of a Protective B Cell Epitope on Outer Surface Protein C (OspC) of the Lyme Disease Spirochete, Borreliella burgdorferi
Source: mBio. 2023 Mar 28;14(2):e02981-22. doi: 10.1128/mbio.02981-22 (PMC10128040; doi:10.1128/mbio.02981-22)

**A**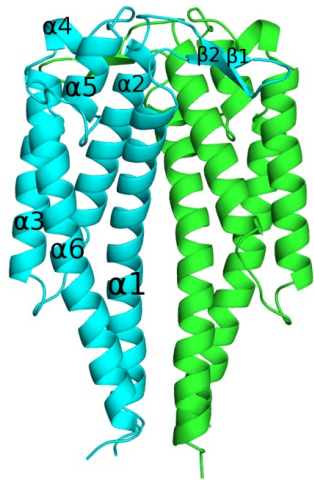

OspC<sub>A</sub>

**B**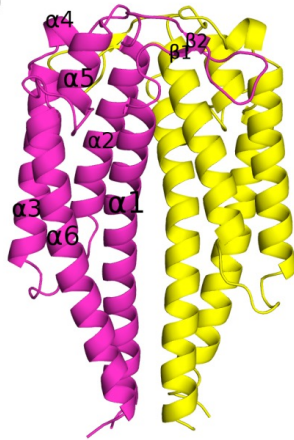

OspC<sub>I</sub>

**C**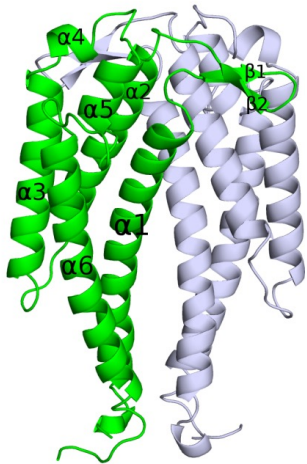

OspC<sub>E</sub>

Supplement: FIG S1 [file mbio.02981-22-s0002.pdf]

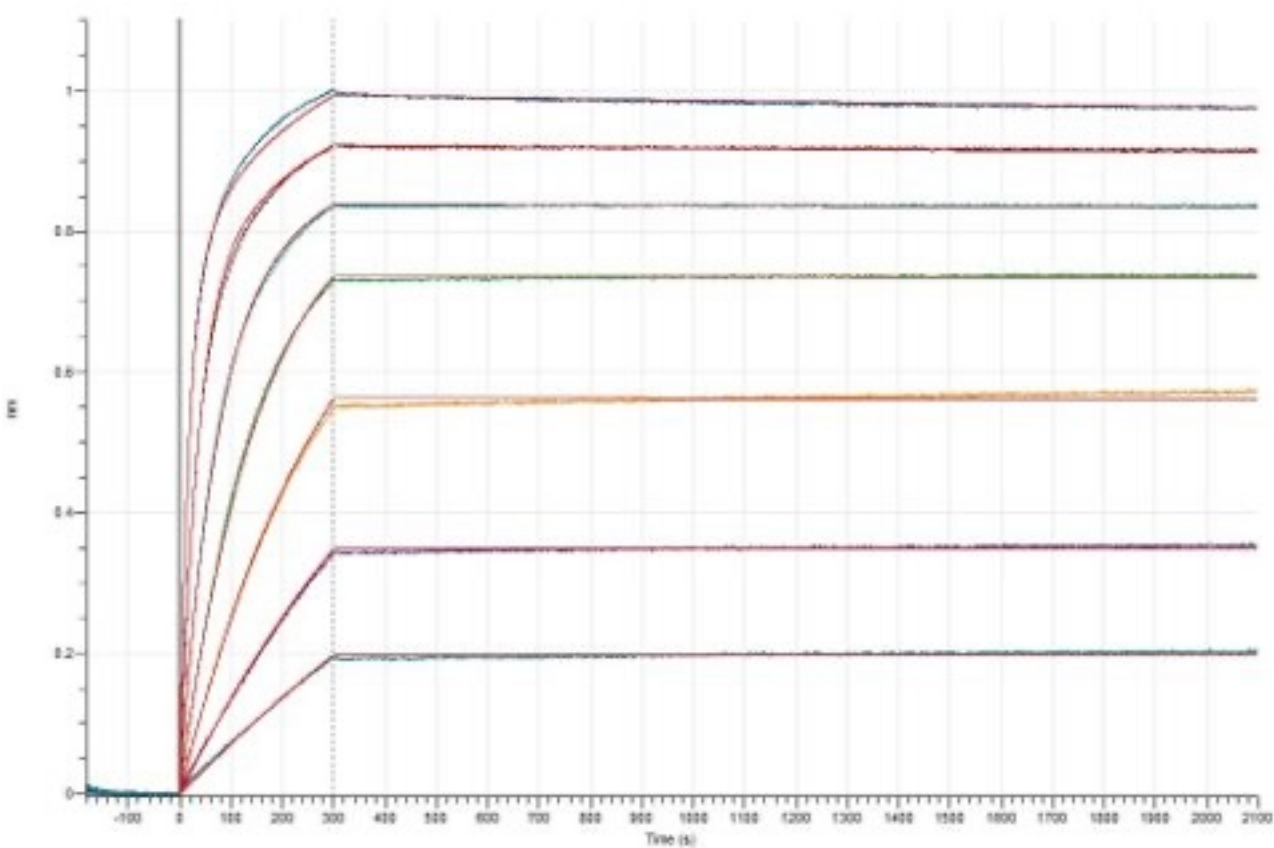

Supplement: FIG S2 [file mbio.02981-22-s0003.pdf]

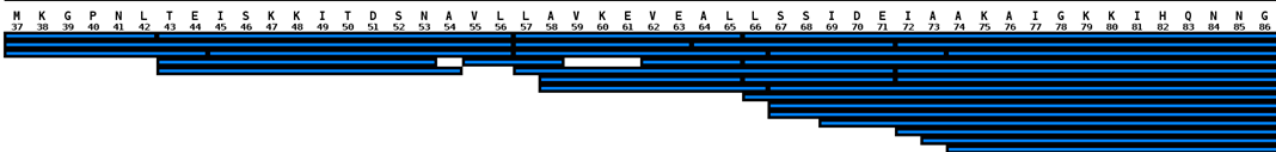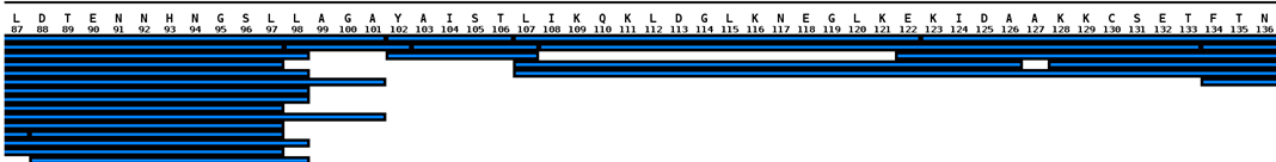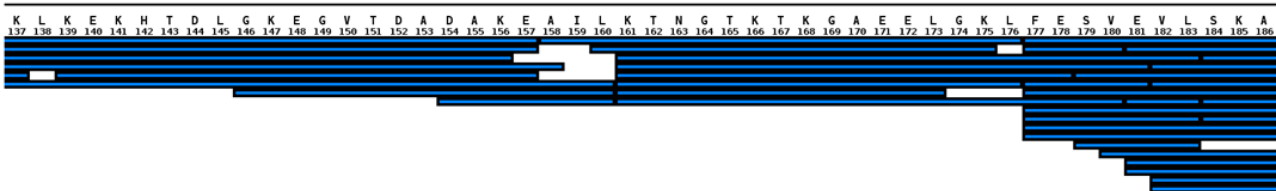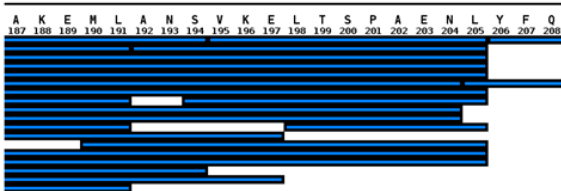

pepsin: 172 of 172 ~ 100%  
Total: 172 of 172 ~ 100%

Supplement: FIG S3 [file mbio.02981-22-s0004.pdf]

**A****OspC<sub>B</sub>**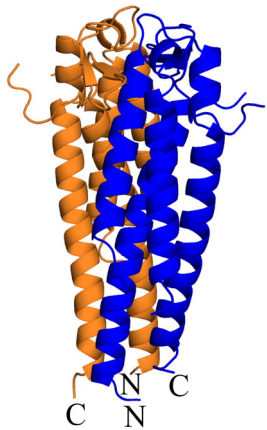**B****OspC<sub>K</sub>**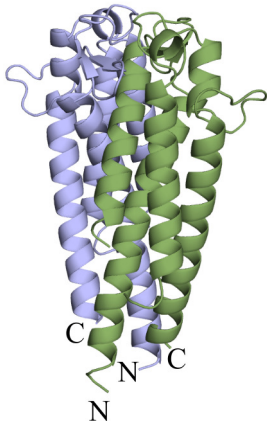**C**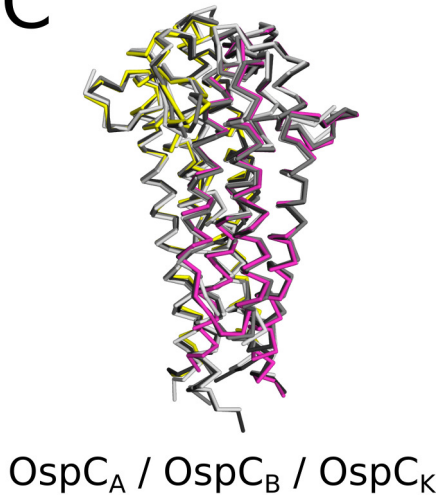

Supplement: FIG S4 [file mbio.02981-22-s0005.pdf]

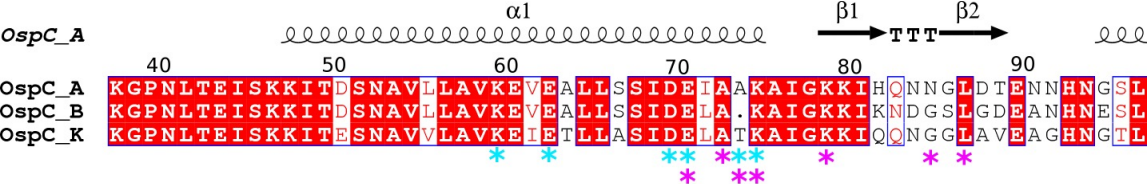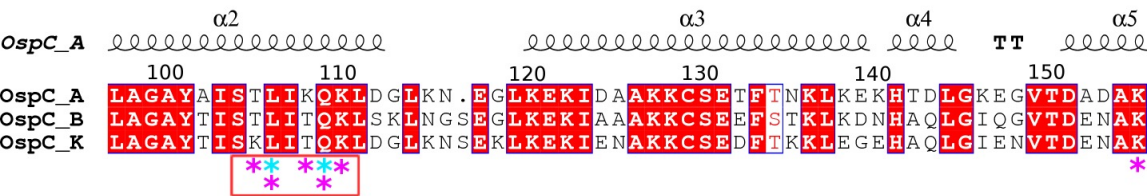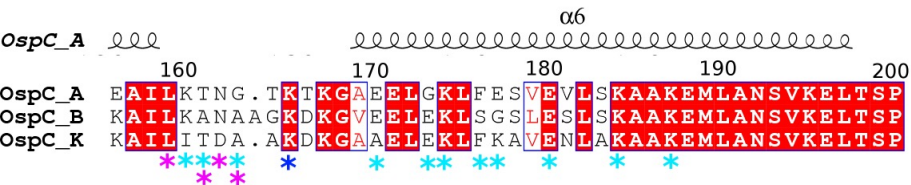

Supplement: FIG S5 [file mbio.02981-22-s0006.pdf]
