# Supplementary material for: Structural Elucidation of a Protective B Cell Epitope on Outer Surface Protein C (OspC) of the Lyme Disease Spirochete, Borreliella burgdorferi
Source: mBio. 2023 Mar 28;14(2):e02981-22. doi: 10.1128/mbio.02981-22 (PMC10128040; doi:10.1128/mbio.02981-22)
Supplement: FIG S6 [file mbio.02981-22-s0007.pdf]

```
A          NSADESVKGPNLTEISKKITDSNAVLLAVKEVEALLSSIDEIAAKAIGKKIHQNNGLDTE   60
C3         NSADESVKGPNLAEISKKITESNAVVLAVKEVETLISSIDEIAAKAIGKKIKNDGSLDND   60
I3         NSADESVKGPNLTEISKKITESNAVVLAVKEIETLLSSIDELATKAIGQKIDA-NGLGVQ   59
B          NSADESVKGPNLTEISKKITDSNAVLLAVKEVEALLSSIDELA-KAIGKKIKNDGSLDNE   59
K          NSADESVKGPNLTEISKKITESNAVVLAVKEIETLLASIDELATKAIGKKIQQNGGLAVE   60
           ************:*******:****:*****:*:*::****:* ****:**.  ..*  :

A          NNHNGSLLAGAYAISTLIKQKLDGLK-NEGLKEKIDAAKKCSETFTNKLKEKHTDLGKEG   119
C3         ANHNGSLLAGAYAISTLITQKLGGLKNSEELKEKIEEAKKCNKAFTEKLKSSHAELGKQD   120
I3         ADQNGSLLAGAYAISTLITQKLSALN-SENLKEKVAKVKKCSEDFTNKLKNGNAQLGLAA   118
B          ANRNESLLAGAYTISTLITQKLSKLNGSEGLKEKIAAAKKCSEEFSTKLKDNHAQLGIQG   119
K          AGHNGTLLAGAYTISKLITQKLDGLKNSEKLKEKIENAKKCSEDFTKKLEGEHAQLGIEN   120
            .:* :******:**.**.***. *: .* ****:  .***.: *: **:  :::**

A          VTDADAKEAILKTNG-TKTKGAEELGKLFESVEVLSKAAKEMLANSVKELTSPVVAESPK   178
C3         AQDDDAKKAILRTHN-TKDKGAEELDKLFKAVENLSKAAKEMLSNSVKELTSPVVAET--   177
I3         ATDADAKEAILKTNG-TKTKGAEELGKLFESVEVLSKAAKEMLANSVKELTSPVVAES--   175
B          VTDENAKKAILKANAAGKDKGVEELEKLSGSLESLSKAAKEMLANSVKELTSPVVVESPK   179
K          VTDENAKKAILITDA-AKDKGAAELEKLFKAVENLAKAAKEMLANSVKELTSPIVAES--   177
           . * :**:*** :.   * **. ** **  ::* *:*******:*********:*.*:
```
