# Supplementary material for: Structural Elucidation of a Protective B Cell Epitope on Outer Surface Protein C (OspC) of the Lyme Disease Spirochete, Borreliella burgdorferi
Source: mBio. 2023 Mar 28;14(2):e02981-22. doi: 10.1128/mbio.02981-22 (PMC10128040; doi:10.1128/mbio.02981-22)
Supplement: TABLE S1 [file mbio.02981-22-s0001.pdf]

**Table S1. Data associated with crystal structures reported in this study.**			
**Data Collection**			
Complex	$OspC_A$-B5	$OspC_B$	$OspC_K$
Space group	$P2_12_12$	$P2_1$	$P2_1$
Cell parameters: *a,b,c* (Å) / β (°)	54.7 / 139.8 / 205.7	42.9 / 44.1 / 68.8 / 103.8	52.3 / 52.3 / 116.7 / 92.5
BNL Beamline	21-ID-E	21-ID-E	21-ID-E
Resolution range ^{a,}(Å)	50-2.70 (2.75-2.70)	50-1.50 (1.53-1.50)	50-1.95 (1.98-1.95)
wavelength (Å)	0.979	0.979	0.979
No. of reflections	3988365	3312121	2685656
Average redundancy^a	2.8 (2.7)	2.7 (2.4)	2.8 (2.6)
$(I)/(\delta)^a$	21.1 (1.2)	31.5 (3.2)	17.6 (1.7)
Completeness^a (%)	99.6 (99.6)	95.8 (95.6)	97.8 (99.2)
$R_{merge}{}^{a,b}$ (%)	8.3 (145.0)	5.9 (32.5)	14.9 (131.1)
$CC_{1/2}{}^{a,c}$	(0.50)	(0.93)	(0.54)
**Refinement**			
Bragg spacings ^{a,}(Å)	49.4-2.7 (2.76-2.70)	36.8-1.5 (1.54-1.50)	39.7-1.95 (1.99-1.95)
R^d / $R_{free}{}^e$ (%)	20.4 / 25.6	14.7 / 16.7	19.3 / 22.8
No. of Protein atoms	8825	4908	4708
No. of Waters	32	270	358
RMSD bond length (Å)	0.005	0.012	0.003
RMSD bond angle (°)	0.78	1.14	0.52
Ramachandran favored / allowed^f (%)	94.8 / 99.8	97.4 / 100	97.0 / 100
PDB code	**7UIJ**	**7UJ2**	**7UJ6**

^{a} Values in outermost shell are given in parentheses.
^{b} $R_{merge} = (\sum | I_i - <I_i> |)/\sum | I_i |$, where I_i is the integrated intensity of a given reflection.
^{c} $CC_{1/2}= (1+q^2\sigma_\varepsilon{}^2/<I>2^{-1}$, where σ_ε denotes the mean error within a half-datase, $CC_{1/2}$ is the correlation coefficient of two split data sets each derived by averaging half of the observations for a given reflection.
^{d} $R = \sum | |F_o| - |F_c| | /\sum | |F_o| |$, where F_o and F_c denote observe and calculated structure factors, respectively.
^{e} R_{free} was calculated using 5% of data excluded from refinement.
^{f} Calculated using Molprobity.
